# Companion pigs alternate orientation between humans and snout-inaccessible targets

**DOI:** 10.1098/rsos.242178

**Published:** 2025-08-27

**Authors:** Paula Pérez Fraga, Fanni Lehoczki, Attila Andics

**Affiliations:** ^1^ Neuroethology of Communication Lab, Department of Ethology, Eötvös Loránd University, Budapest, Hungary; ^2^ Department of Ethology, Eötvös Loránd University, Budapest, Hungary; ^3^ NAP Canine Brain and Tissue Research Group, Department of Ethology, Eötvös Loránd University, Budapest, Hungary

**Keywords:** dog, pig, referential communication, interspecific communication

## Abstract

Previous research on intensely human-socialized pigs found no evidence for orientation alternations to direct human attention to an inaccessible target, the benchmark behaviour that in many other species has been reported to evidence capacity for functionally referential communication with humans. However, the unsolvable task paradigms that are typically used may mask communicative capacities by promoting manipulative behaviours in animals with strong independent problem-solving tendencies, like pigs. Here, using a novel out-of-reach paradigm that does not induce manipulative biases, we reassessed the capability of pigs for functionally referential communication with humans. We compared the emergence of orientation alternations between a human and an elevated, physically inaccessible target in adult companion pigs and dogs, a species characterized by more human-dependent problem-solving. We found that with these settings, pigs attempted to solve the task independently even less often than dogs and, similarly to dogs, pigs also exhibited orientation alternations. This is the first report demonstrating human-oriented functionally referential communicative behaviours in pigs, suggesting that this capacity may be more widespread across mammals than previously thought.

## 1. Introduction

Referential communicative behaviours, which direct the social partner’s attention to specific targets, are key in human social cognition [1,2]. Human-oriented functionally referential communicative capacities have been evidenced not only in domestic animals [3–5] but also in wild species [6,7]. But recent reports on intensely human-socialized pigs’ failures to engage in functionally referential communication with humans—despite using task settings comparable to those used with other species, including similarly kept dogs—raised questions about the prevalence of such capacities among mammals [8,9].

Commonly used paradigms to elicit human-oriented referential communication in animals include unsolvable tasks [6,8,10] and out-of-reach scenarios [5,11,12]. In unsolvable task scenarios, a previously working manipulative solution to access a reward stops working, whereas in the out-of-reach scenarios, a reward-hiding place, inaccessible to the animal, turns out to be accessible for humans. The assumption in all cases is that the tasks will prompt the test animal to direct the human’s attention to the inaccessible reward through various behaviours, like gaze alternations between the human and the target. Gaze alternation, in particular, is often regarded as a hallmark of functional referential communication [4,6,7].

Pigs, despite displaying some human-oriented behaviours comparable to those of similarly socialized dogs in similar contexts [13], have not shown an increase in gaze alternation behaviour when faced with and impossible-to-get reward compared with a baseline in which no reward was present during an unsolvable task paradigm [8]. Similarly, they have not displayed orientation alternations between a human and a desirable reward that was placed out of reach in a manipulative task, such as inside of a box on the floor [9]. At least two possible explanations could account for the lack of human-oriented communicative behaviours with referential properties in pigs, specifically, orientation alternations between the target and the human present. On the one hand, these results may indeed reflect pigs’ inability to direct human attention to a specific target, perhaps caused by their reduced visual communicative capacities [14–17]. Alternatively, it may be the task setting that triggers pigs’ natural independent foraging strategy, which relies heavily on physical manipulation, specifically on rooting the ground [18,19]. In this latter case, the previously used experimental arrangements (unsolvable task scenarios and an out-of-reach task where the reward was hidden in a box at floor level) may have enhanced pigs' manipulative, independent problem-solving behaviours, overshadowing any inclination to communicate with humans.

To disentangle these hypotheses, here we assessed animals’ human-oriented functionally referential communication, using a novel, not only unsolvable but also snout-inaccessible task where physical access to the target was disabled by raising it above the animals’ reach. Previous out-of-reach tasks on elevated surfaces succeeded in revealing human-oriented referential communicative behaviours also in species (e.g. wolves) [11] that failed to exhibit such behaviours in manipulative unsolvable tasks [20], probably because of being highly persistent in attempting to obtain the reward on their own, and thus continuously interacting with the apparatus. Here, our out-of-reach task was specifically designed to clearly discourage pigs’ natural snouting strategy, but at the same time kept them focused on the reward by gradually elevating it to a height inaccessible by snout.

Therefore, we predicted that if the lack of human-oriented, functional referential behaviours previously found in pigs was merely a side effect of their bias for manipulative, independent problem-solving, then pigs would show such behaviours in the current non-manipulative out-of-reach task. Conversely, if previous findings truly reflect pigs’ limited capacity for functional referential communication, then pigs would fail to perform orientation alternations in the present task as well. We compared the behaviours of adult companion pigs to those of dogs. Dogs, having been selected for efficient cooperation with humans, are more human-dependent problem solvers for whom this capacity has been widely reported.

## 2. Material and methods

### 2.1. Subjects

We recruited adult family pigs (*n* = 18) and dogs (*n* = 28) from Hungary and Spain whose owners applied to participate through an online form. In the final analysis, we included 14 pigs: seven males (neutered) and seven females (6 neutered and one intact), aged between 2 and 8 years (mean ± s.d. = 4.55 ± 1.42 years). Pig breeds included Minnesota miniature pigs and their mixes, along with two Vietnamese pot-bellied and one Juliana miniature pig (see electronic supplementary material, table S1 for subject details). We included 18 dogs, 8 males (neutered) and 10 females (neutered), aged between 2 and 13 years (mean ± s.d. = 5.90 ± 3.42 years) from 7 mixed breeds and 11 purebreds (from 6 breeds).

Each subject participated in a single testing occasion and only those tests considered ‘appropriate’ were included in the analysis (for a detailed description of the test, see the ‘procedure’ section). Appropriateness was determined as follows: (i) the owner carefully following the provided protocol step by step (e.g. setting up the room under the description provided, performing all the steps, etc.); (ii) absence of environmental distractions (e.g. loud noises, etc.); (iii) the subject not spending more than 20% of the total time out of the camera’s view; and (iv) the subject not carrying the container in its mouth, etc. Tests that did not meet the inclusion criteria were excluded from the analysis. This excluded four pigs (one due to camera misplacement, and three that spent over 20% of the time out of frame) and ten dogs (three that played with and carried the container, three that spent over 20% of the time out of frame and four with procedural issues such as poor camera angles or early release by the owner).

### 2.2. Set-up

Similarly to a previous citizen science study [21] we conducted, the owners were provided with a document containing: (i) a description of the main aim of the study; (ii) instructions for preparing the testing room; (iii) a step-by-step description of the behavioural test; (iv) links to the demonstration video material; and (v) a data protection note.

The required elements for the behaviour test were: (i) at least one video recorder device (smartphone, tablet, or camera); (ii) a laptop/PC with an internet connection and video conferencing capability, as the test was conducted through Zoom with the experimenters; (iii) the owner (O) and another human helper (H) who was familiar to the animal; (iv) a plastic container (which was not the subjects’ own food bowl and in which the animal could easily insert its snout or muzzle and retrieve the food from the bottom); (v) pieces of high-value treats; (vi) *Object-1* (chair, short table, etc.) adjusted to the size of the subject, with a height such that the animal could reach the container positioned on it and eat the food from it; and (vii) *Object-2* (table, shelf, etc.) also adjusted to the size of the subject, with a height such that the animal could not reach the container positioned on it (ensuring there was not a large difference between *Object-1* and *Object-2*).

The test was conducted in a familiar, spacious room in the O’s house. The centre of the room, marked by the O (e.g. with tape), was designated as the location for the plastic food container. From this mark (food container position), the O measured a 1 m distance and placed a second marker, which indicated the location of the O during the entire test. From the food container location, the O measured another 1 m, perpendicular to the O’s location and placed a third marker, which indicated the location of the H. The camera was positioned in front of the setup, aiming to capture as much space as possible. The video chat device (usually a laptop) could be positioned at a different angle from the camera to include the whole room, or in the same place if that location provided a full view.

**Figure 1.**
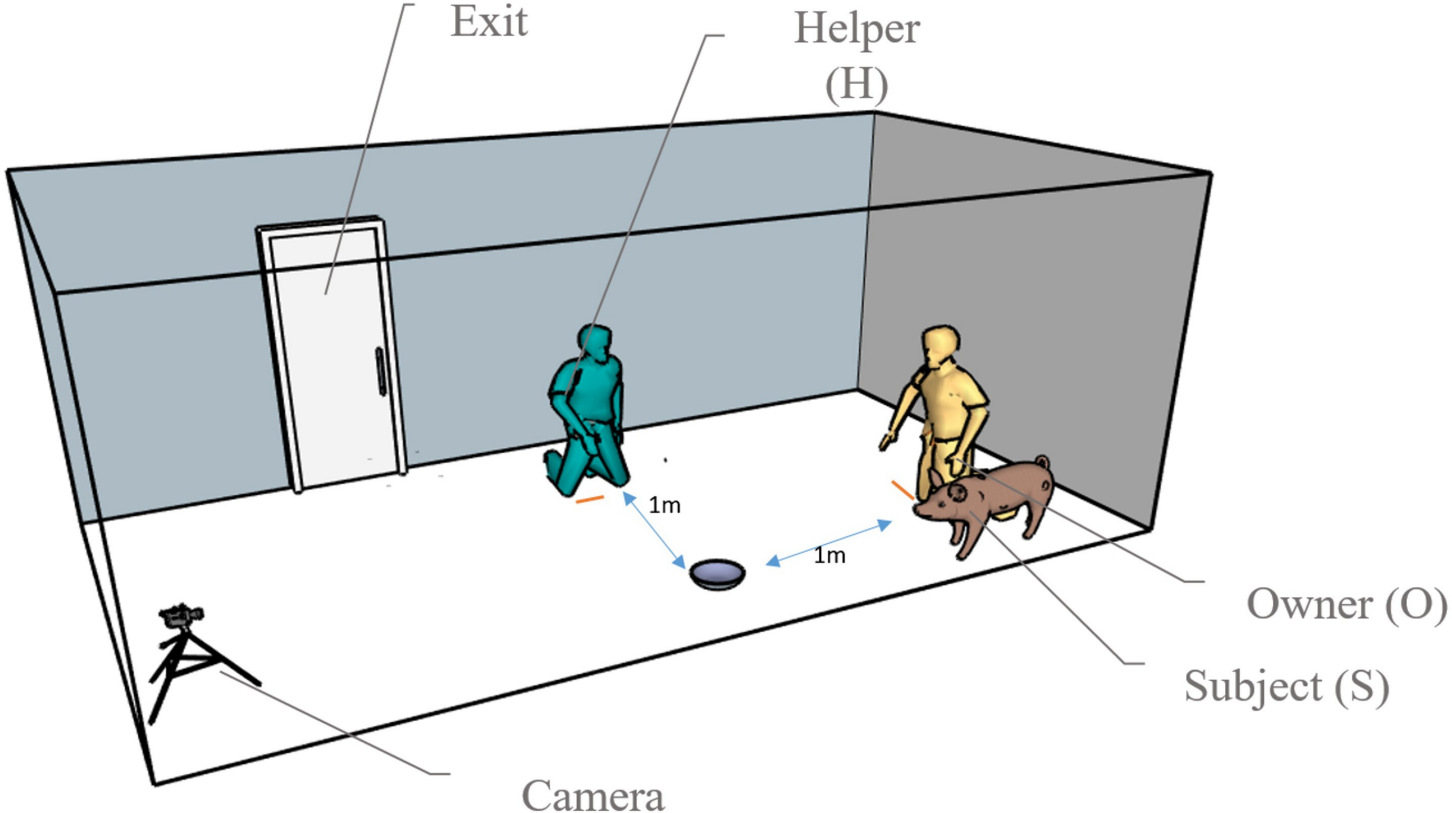
Illustration of the room set-up of the behaviour test, with the camera positioned in the same place as the videochat device would be. The O and the H positions were at 1 m of distance from the plastic box location (perpendicular to each other). The subject moved freely in the room while the camera and the videochat device recorded its behaviour*.*

Figure 1 shows an ideal setup of the room (the chat device would be at the same location as the camera). The O was required to store the pieces of the high-value food in an adjacent room, accessible from the testing room. The test required minimal outside disturbances, and only the O, H and subject (S) were allowed in the room with the exit closed.

### 2.3. Procedure

After setting up the testing room, the O started a video call with one of the experimenters (E) who double-checked the setup and allowed time for the O to ask any remaining questions. Afterwards, the H started the recording from the recording device and the E began recording the Zoom meeting. The behaviour test consisted of four short consecutive parts (a modified version of that described previously [8]): (i) no-food condition (1 trial, 2 min) (for observing any baseline human or apparatus-oriented behaviours in the absence of food and for familiarization with the setup); (ii) Reachable-1 condition (3 trials, max 1 min each) (food is on the floor level); (iii) Reachable-2 condition (3 trials, max 1 min each) (food is on *Object-1*, elevated but reachable); and (iv) out-of-reach condition (1 trial, 2 min) (food is on the top of *Object-2*, visible but unreachable). The E set the start moment of the behaviour test. The main rationale of the test was to conserve the structure of the previous unsolvable task.

During all conditions, the O and the H either kneeled or sat cross-legged at their designated locations, facing forward with their arms crossed in front of their bodies. At the beginning of every condition, the O kept the S next to him/her by gently holding him/her from behind or by the harness, by petting him/her, or by placing an arm in front of the S, facing the container location. In the no-food condition, the H stood up, manipulated the container, grabbed the S’s attention (clicking tongue, calling by name, etc.), imitated placing a piece of food into the container and shook it. The H then placed the container on the floor, in its location and kneeled at his/her mark. The O said ‘yours’ (or another command used for this situation) (this was performed to ensure symmetrical procedures in both the no-food and the out-of-reach condition) and let the S free to explore the container (and the room) for 2 min. The E marked the end of the condition with a sound signal through the video chat. The purpose of the no-food condition was to observe the emergence of any behaviours directed towards the container and the human in the absence of food, serving as a baseline for comparison with the behaviours observed during the out-of-reach condition. This was a similar rationale to that used in our previous study using an unsolvable task paradigm [8].

The test continued with the Reachable-1 condition after the O and the S returned to their starting positions. The H brought a piece of food from the other room, grabbed the container, called the S’s attention and placed the piece of food into the container, ensuring that the S was watching. The H then shook the container, placed it on the floor on its location and kneeled at his/her mark. O said ‘yours’ and let the S free to eat the food from the container. The trial lasted until the dog/pig ate the food or until 1 min had passed, which the E marked with a sound. H and O could praise S and then they returned to the starting position. The Reachable-1 condition was repeated three consecutive times. The purpose of this condition was to inform the subjects that food was involved and to motivate them to obtain it. After the third trial of the Reachable-1 condition, the Reachable-2 condition started. The H placed *Object-1* at the container’s marked spot, and then put the container on top. The O and the S returned to their starting position. The procedure was the same as in the Reachable-1 condition, with the only difference being that the H placed the container on *Object-1* each time, instead of on the floor. This condition was repeated three times. The primary aim of the Reachable-2 condition was to motivate the S to look for the food at a height. The purpose of including the Reachable conditions after the no-food condition was twofold. First, to allow the animals to understand that a food reward was available and to motivate them to obtain it. Second, by gradually increasing the height of the reward, we aimed to ensure that the subjects maintained focus on the reward and were aware that it was positioned at an elevated location, so they would remain motivated to search for it in the out-of-reach condition when it was impossible to obtain.

**Figure 2.**
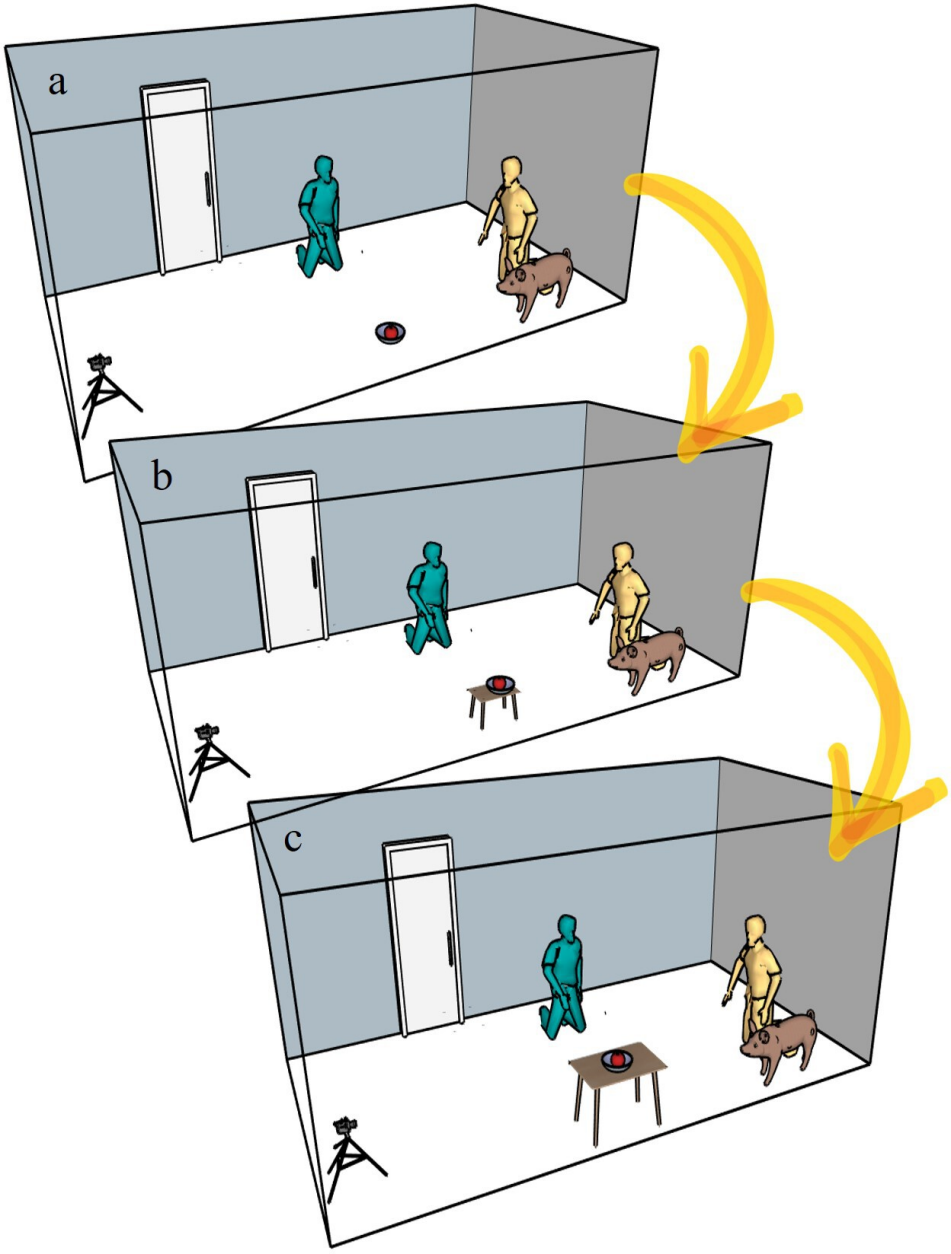
Illustration of the Reachable-1 condition (*a*), Reachable-2 condition (*b*) and out-of-reach condition. The subject moved freely in the room while the camera and the videochat device recorded its behaviour*.*

For the last out-of-reach condition, the H replaced *Object-1* with *Object-2* and placed the container on top of it. The O and the S returned to their starting position. Similarly to the reachable conditions, the H brought a piece of food, called the S’s attention, placed the food into the container, shook it, and placed the container on *Object-2*, where it was unreachable for the S. The H kneeled at his/her mark, the O said ‘yours’ and let the S free for 2 min. The H and the O remained looking forward with their arms crossed. The E marked the end of the 2 minutes (and the end of the test) with a sound signal. The H and the E stopped recording, and the O was allowed to give the food to the S (see figure 2 for an illustration of the conditions). Finally, the O was asked to send the video from their recording device. All owners who participated were provided with a certificate.

### 2.4. Video analysis

Behaviour analysis of the videos was performed using BORIS (Behavioural Observation Research Interactive Software, version 8.25.4), starting when the O released the S in all conditions and ending when the researcher marked the end of the test (2 min for both no-food and out-of-reach conditions). In the Reachable-2 condition, we measured the latency to success (access the food). In the no-food and out-of-reach conditions, we measured the duration of orientation and interaction with humans and the container (i.e. target) (during the out-of-reach condition, as the plastic container was impossible to manipulate, we included the orientation towards and interaction with *Object-2*). We also assessed the frequency of vocalizations (including human/container-oriented vocalizations), attempts to solve the task (i.e. access the plastic container) (only in the out-of-reach condition) and alternations of orientation between the target and humans. This was measured using two elements (i.e. human–apparatus or vice versa, with a gap of less than 2 s between them) and three elements (i.e. human–apparatus–human or vice versa) with a gap of less than 2 s between each element; see electronic supplementary material, table S2 for detailed definitions of behavioural variables). Two experimenters coded all videos, and a secondary coder, blind to the study’s aim, independently coded 20% of them. Coding reliability was determined using kappa statistics, yielding an average kappa of 0.684 ± 0.07, which is considered a moderate to good level of agreement [22].

### 2.5. Statistical analysis

We analysed the data using R statistical environment (RStudio version 2023.09.0 with R version 4.6.3). To compare baseline human-oriented and container-oriented behaviours in the absence of a reward with similar behaviours in the presence of a desirable but unreachable reward (following a similar procedure to a previous study [23]), we assessed the main effects of species and condition (no-food and out-of-reach), and their interaction, including the subject ID as a random factor. For analysing the frequency of alternation of orientation, frequency of vocalizations and the number of attempts to solve the task, we used generalized linear mixed models (GLMMs) with Poisson distribution and log-link function (lme4 package). The proportion of vocalization emitted while orienting towards the human or container was analysed using nonparametric beta regression (glmmTMB package). For duration data, we used the Shapiro–Wilk test and normal Q–Q plots to assess the distribution of the response variables and residuals. Where necessary, we applied Tukey transformations with optimal lambda parameters to fulfil normality criteria. Variables that met the normality assumption (e.g. orientation to humans and box) were analysed using linear mixed-effects models. Duration of interaction with humans and box did not fit normality after transformation, and we opted for transform them in ratios. We analysed them using nonparametric beta regression (glmmTMB package). Finally, we analysed subjects' latency to obtain the reward in the Reachable-2 condition using Cox regression (‘coxme’ package) (we assessed the main effects of species, trial and their interactions).

## 3. Results

### 3.1. Latency to access the reward during the Reachable-1 condition

All dogs ate the food in all three Reachable-1-condition trials. Only two pigs did not eat the food in the first trial, but all pigs ate the food in the subsequent trials. Furthermore, we analyse the latency to retrieve the reward in the Reachable-1 condition to assess the motivation of the subjects. Trial and species affected the subjects’ success latency in getting the reward (Cox regression, LRT: χ^21^ = 90.47, *p* < 0.001). Subjects improved significantly in trials 2 and 3 compared with trial 1 (*p* = 0.008 and *p* < 0.001, respectively), with no significant difference between trials 2 and 3 (*p* = 0.494). Overall, dogs were faster than pigs (β ± s.e. = −3.57 ± 0.73, Z = −4.91, *p* < 0.001) (see electronic supplementary material, table S3). Species differences found here likely reflect species-specific speed rather than problem-solving abilities.

### 3.2. Latency to access the reward during the Reachable-2 condition

All subjects retrieved and ate the food reward in all trials of the Reachable-2 condition. Furthermore, on the Reachable-2 condition, both trial and species affected the subjects’ success (Cox regression, LRT: *χ*^2^ = 22.625, *p* < 0.001). Subjects improved significantly in trials 2 and 3 compared with trial 1 (*p* = 0.007 and *p* < 0.001) without differences between trials 2 and 3 (*p* = 0.428). Overall, dogs were faster than pigs (*β* ± s.e. = − 3.449 ± 0.66, *Z* = −5.16, *p* < 0.001) (see electronic supplementary material, table S4).

### 3.3. Frequency of alternation of orientation

**Figure 3.**
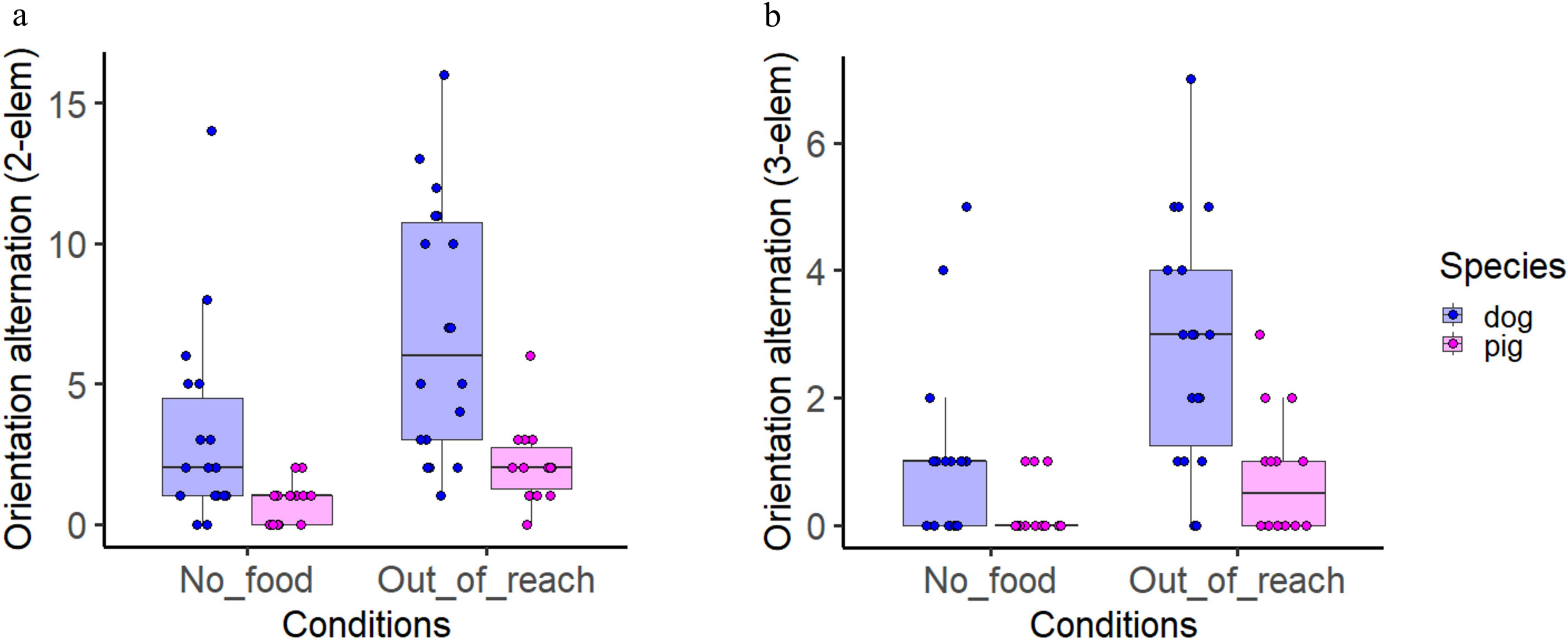
Frequency of alternation of orientation using a two-element sequence (*a*) and a three-element sequence (*b*) for both species. Boxplots indicate the median 25th and 75th percentiles (boxes), and the minimum and maximum (whiskers). Dots represent individual data points.

Species and condition had significant effects on the frequency of alternations of orientation in a two-element sequence (GLMM, LRT: *χ*² (1) = 16.11, *p* < 0.001 and LRT *χ*² (1) = 35.193, *p* < 0.001). Subjects alternated orientation more in the out-of-reach condition than in the no-food (*Z* = 5.687, *p* < 0.001), and dogs oriented more frequently than pigs across both conditions (*Z* = −4.464, *p* < 0.001; figure 3a). Similar results were observed with a more restrictive three-element sequence (main effect on species, GLMM, LRT: *χ*² (1) = 15.204, *p* < 0.001 and condition, LRT: *χ*² (1) = 21.169, *p* < 0.001), where dogs and pigs both alternated more in the out-of-reach condition (*Z* = 4.288, *p* < 0.001), with dogs again showing higher frequency than pigs (*Z* = 4.288, *p* < 0.001; figure 3b). To ensure comparability with previous studies, [8] we also analysed the two-element sequence within the first minute of the condition and found consistent results with the total condition duration, indicating that extra time did not significantly affect performance (GLMM, species LRT: *χ*² (1)10 = 10.671, *p* < 0.001 and condition, LRT: *χ*² (1) = 21.126, *p* < 0.001, out-of-reach (*Z* = 4.430, *p* < 0.001) and dogs more than pigs (*Z* = −3.486, *p* < 0.001; see electronic supplementary material, tables S5–S7 for complete model results).

### 3.4. Frequency of vocalizations

Since only 3/18 dogs vocalized in the no-food condition and 5/18 in the out-of-reach condition, we analysed only the pigs, as 12/14 vocalized in the no-food condition and 10/14 in the out-of-reach condition. Condition significantly affected the frequency of pig vocalizations (GLMM, LRT: *χ*² (1) = 25.11, *p* < 0.001), with pigs vocalizing more in the no-food than in the out-of-reach condition (*Z* = −4.906, *p* < 0.001). However, condition did not influence the proportion of vocalizations directed towards the humans or the container (see electronic supplementary material, table S8 for complete model results).

### 3.5. Attempts to solve the task

**Figure 4.**
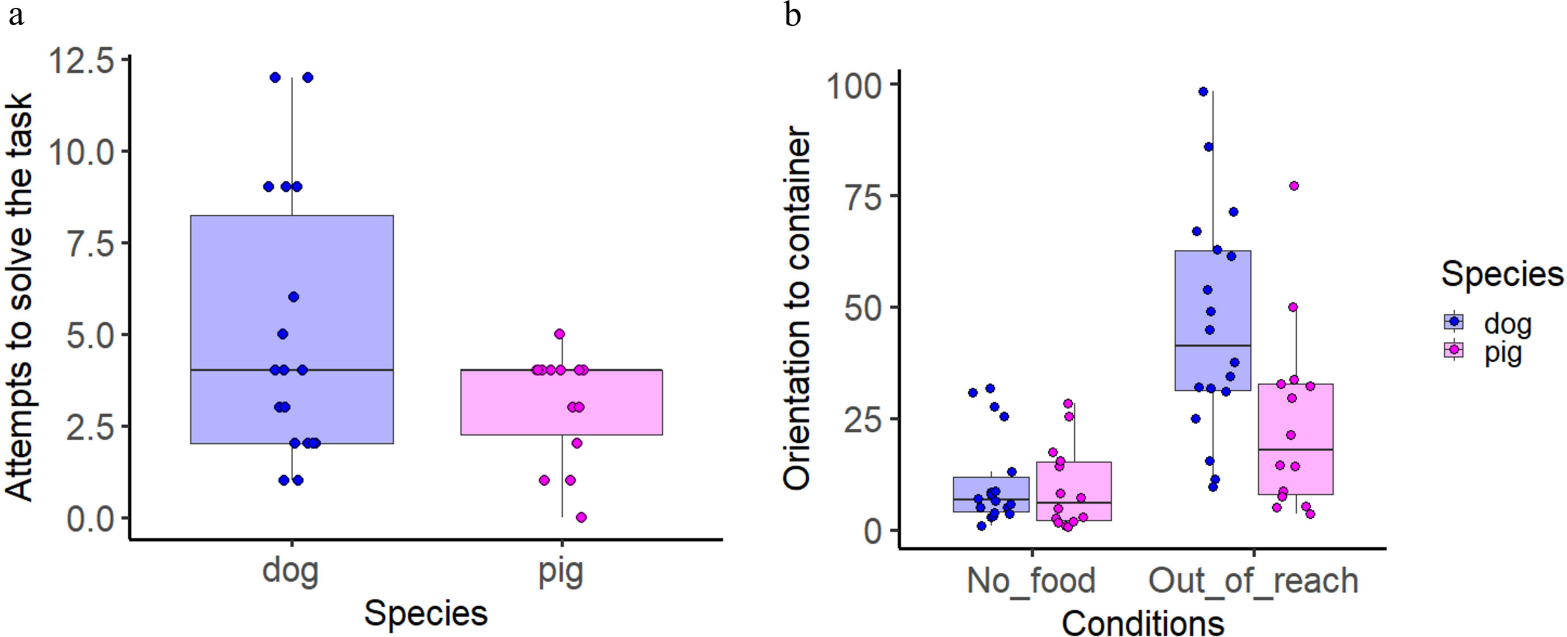
Number of attempts to solve the tasks during the out-of-reach condition for both species (*a*) and time spent orienting towards the container for both species (*b*). Boxplots indicate the median 25th and 75th percentiles (boxes), and the minimum and maximum (whiskers). Dots represent individual data points.

The factor species showed a trend towards affecting the frequency of attempts to solve the task (i.e. getting the food) (GLMM, LRT: *χ*² (1) = 3.35, *p* = 0.058), with dogs tending to attempt more often than pigs (*Z* = −1.891, *p* = 0.058; figure 4a; see electronic supplementary material, table S9 for complete model results). For clarification, the main idea behind the out-of-reach condition was that the food was inaccessible and therefore, no subjects were able to get the reward.

### 3.6. Orientation to humans and container

Neither species nor condition significantly affected the duration of orientation towards humans. However, the duration of orientation towards the container showed a tendency effect towards interaction between species and condition (LMM, LRT: *χ*² (1) = 2.78, *p* = 0.09). Post hoc comparisons revealed that both dogs and pigs oriented more towards the container in the out-of-reach condition compared with the no-food condition (*p* < 0.001, *p* = 0.001). Additionally, in the out-of-reach condition, dogs oriented more towards the container than pigs (*p* = 0.009; figure 4b; see electronic supplementary material, tables S10–S12 for complete model results, post hocs and comparison of model performances).

### 3.7. Interactions with humans and container

Species and condition influenced the duration of physical interactions with humans (GLMM, species LRT: χ² (1) = 4.1384, *p* = 0.041 and condition, LRT: χ² (1) = 5.627, *p* = 0.017), with pigs showing a stronger inclination to interact physically with humans compared with dogs (*Z* = 2.065, *p* = 0.038), and both species interacting more with humans during the no-food condition (*Z* = −2.518, *p* = 0.011). However, neither species nor condition significantly affected physical interactions with the container (i.e. *Object-2* in the out-of-reach condition) (see electronic supplementary material, table S13 for complete model results).

## 4. Discussion

Using a novel non-manipulative out-of-reach task, we assessed the emergence of human-oriented communicative behaviours with referential properties in companion pigs and dogs. In this paradigm, unlike in previous ones, pigs performed more orientation alternations between the target and the humans during the out-of-reach condition compared with the no-food condition, even if to a lesser extent than dogs. Furthermore, although both species oriented more towards the target during the out-of-reach condition, dogs did so more than pigs, opposite to previous studies where pigs were more food-focused [9]. Relatedly, pigs in the present study were not less owner-oriented than dogs when exposed to the out-of-reach reward. Interestingly, although both pigs and dogs improved across the different trials of the reachable conditions, indicating that all animals remained motivated to obtain the reward, dogs were generally faster than pigs. This was true not only when retrieving the reward from floor level, which may simply reflect higher overall speed in this species, but also when retrieving the elevated but still reachable reward. Additionally, dogs made more attempts than pigs to acquire it independently once it became unreachable. This contrasts with previous findings [8], suggesting that the current experimental settings have significantly influenced pigs' problem-solving strategies, revealing a performance unseen with typically used paradigms. Overall, this work provides the first evidence that pigs, just as many other researched species, can functionally referentially communicate with humans.

This study provides novel evidence that pigs, just like dogs, use orientation alternations to seemingly direct the attention of humans to an out-of-reach target. This behaviour proved robust, with increased occurrence not only for two-element sequences (i.e. a single alternation between a human and a target; as usually measured in similar paradigms [8,9,12,24,25] but also for more demanding three-element sequences (i.e. two consecutive alternations: target–human–target or human–target–human). Such consecutive alternations of orientation strengthen this behaviour’s potential to attract the attention of the partner and focus it on the target, and have long been interpreted as evidence for functional referentiality in other species [7,10,26]. This finding is particularly significant as it reveals that pigs can also use visual communicative signals efficiently, despite their limited use of these in intraspecific communication and their poorer performance in visual communicative tasks with humans [27] (such as following human pointing [13,28,29] or other out-of-reach tasks [9]) compared with other, more visual species, such as dogs [30].

What can be the reason for finding functionally referential communication in pigs in the present study but not with previous paradigms? We propose that the experimental settings used here were key to demonstrating this capacity in pigs. Indeed, unlike previously used paradigms, the novel, non-manipulative out-of-reach task appears to have changed pigs' typical independent performance during problem-solving. The apparent impossibility to even attempt to access the food through snout manipulation may have prompted pigs to switch strategy and display such human-oriented visual communicative behaviours. Another, not mutually exclusive explanation for our results related to the task design is the potential reduction in the visual and olfactory salience of the reward compared with previous studies, where the reward was placed at floor level and more easily perceived. This higher salience in earlier tasks may have made it harder for pigs to inhibit their direct response in trying to get the reward. In the current study, although the reward was positioned above the pigs’ reach, possibly reducing visibility, it remained olfactorily accessible due to the open plastic bowl, with olfaction being the pigs’ most developed sense [17,31]). While we cannot entirely rule out the role of reward salience, we suggest that the pigs’ inability to access the food using natural foraging behaviours (e.g. rooting or manipulation) may have played a more significant role in reducing task-oriented responses and increasing human-oriented communicative behaviours. Additionally, pigs' age may have weighed in as well; unlike in previous studies that used juvenile animals [8,9,13], here the subjects were adults. Indeed, pigs might require a longer exposure to human communication to display communicative behaviours with referential properties, implying that this ability might emerge later in their development compared with dogs who exhibit these behaviours from an early age [8,32]. Finally, it is possible that the experimental setup, at home with two familiar people, might have enhanced the behaviours observed here, which could also be true for dogs. Both pigs and dogs have been shown to react differently to familiar versus unfamiliar people [23,33], and both species exhibit mild stress responses in unfamiliar or novel environments [34]. Pigs might have felt more comfortable in this setup compared with previous laboratory ones, which could have allowed us to observe the human-oriented-referential communicative behaviours here more easily.

This study also confirms dogs’ superior predispositions for engaging in human-oriented communication [5,35,36], which has long been attributed to their evolutionary selection for cooperation and dependence on humans [37–39]. Unlike box-manipulating tasks, where pigs had a natural advantage over dogs, the current task to reach an elevated target is more natural to dogs than to pigs, supporting a potential switch to independent problem solving in dogs more than in pigs. But even though dogs indeed oriented towards the food more and attempted to solve the task more often than pigs, dogs also displayed more orientation alternations than pigs here, similarly to other paradigms. Thus, even if the naturalness of the task can increase dogs’ motivation, it does not overshadow their inclination to engage in functionally referential communication with humans.

Furthermore, there are behaviours that pigs exhibit more often than dogs but that do not seem to have a referential function. On the one hand, consistent with prior research, pigs were more vocal [8,13] than dogs throughout the whole paradigm. Importantly, however, the proportion of pigs’ human- and apparatus-oriented vocalizations did not increase in the out-of-reach condition, and overall, they vocalized more in the no-food condition. The majority of vocalizations emitted by the pigs during the no- food condition were mostly grunts and, in a few cases, high-pitched grunts. Grunts are the basic call type in pigs, and are non-situation-specific [40], often associated with locomotor activity [41] and linked to exploratory behaviour. Although the increased frequency of grunting, as well as the presence of high-pitched grunts, could indicate increased arousal, the pigs were probably not experiencing high stress during the no-food condition, as evidenced by the absence of vocalizations typically associated with distress, such as squeaks or squeals [42]. We suggest that the potential increase in arousal might instead reflect excitement or interest in the setting, a pattern likely present across all conditions and not unique to the no-food condition. However, since the no-food condition did not involve a specific task to focus on, pigs may have engaged more in exploratory behaviours and consequently, produce more grunts. This supports the idea that the pigs’ vocalizations here may reflect their inner state, rather than the intent to direct the human’s attention. On the other hand, pigs touched humans more often than dogs did but, similarly to vocal behaviours, physical contacts happened more often during the no-food condition. More touches overall in pigs than dogs might simply reflect pigs’ general species-specific predisposition for interacting through physical manipulation [9,18,43], which could also extend to interactions with humans, as suggested by previous research [23,44,45]. The increase of these behaviours in the no-food condition also shows that touching the human may not serve a referential communicative purpose in pigs but rather function simply as the initiation of an interaction. Indeed, although there was no explicit task during the no-food condition, the context itself created a task-like situation for the animals. Thus, having no way of obtaining the target on their own, turning to their owners may have been a reasonable option for these companion animals, who are accustomed to receiving both instructions and rewards from their owners in daily routines.

It has to be noted that the online citizen science approach, while advantageous for testing animals in their homes and increasing sample size and subject variability [21], presented some limitations for this study. The technical devices used for recording, mostly video cameras and laptops, rather than professional laboratory camera systems, may have affected the quality of our observations. Nevertheless, the behaviours included in our ethogram and analyses were clearly visible and properly codable from the videos sent by the owners. While some subtle behaviours might have been missed, our results still offer strong evidence of human-oriented communicative behaviours in both pigs and dogs. Furthermore, although we aimed to use objects to place the reward on that maintained a similar proportion to the size of the subject (i.e. higher elevations for larger animals, and comparable elevations for similar-sized animals), limitations in the available equipment at home may have caused subtle variations in the visibility of the reward in the elevated conditions. However, the fact that both pigs and dogs could retrieve the food during the first elevated condition (Reachable-2) and showed increased apparatus-oriented behaviour during the out-of-reach condition suggests that relative elevation level differences did not significantly impact performance.

To sum up, here we compared the behaviours of companion pigs and dogs in a novel out-of-reach paradigm to demonstrate for the first time that when manipulation of the task is made impossible, even pigs—typically manipulative, independent problem solvers—direct the attention of a human present to the inaccessible reward, similarly to dogs. This revealed that the ability for functionally referential communication is more widespread than previously known. The present findings open up new avenues to assess human-oriented behaviours with referential properties in a range of species with different problem-solving strategies.

## Data Availability

Data and code for this study are available in the electronic supplementary material [46].

## References

[1] Pika S. 2012 The case of referential gestural signaling. Where next?. Commun. Integr. Biol. **5**, 578–582. (10.4161/cib.22012) 23336028 PMC3541325

[2] Liszkowski U. 2011 Three lines in the emergence of prelinguistic communication and social cognition. J. Cogn. Educ. Psychol. **10**, 32–43. (10.1891/1945-8959.10.1.32)

[3] Nawroth C, Brett JM, McElligott AG. 2016 Goats display audience-dependent human-directed gazing behaviour in a problem-solving task. Biol. Lett. **12**, 20160283. (10.1098/rsbl.2016.0283) 27381884 PMC4971169

[4] Malavasi R, Huber L. 2016 Evidence of heterospecific referential communication from domestic horses (Equus caballus) to humans. Anim. Cogn. **19**, 899–909. (10.1007/s10071-016-0987-0) 27098164

[5] Miklósi A, Polgárdi R, Topál J, Csányi V. 2000 Intentional behaviour in dog-human communication: an experimental analysis of 'showing' behaviour in the dog. Anim. Cogn. **3**, 159–166. (10.1007/s100710000072)

[6] McElligott AG, O’Keeffe KH, Green AC. 2020 Kangaroos display gazing and gaze alternations during an unsolvable problem task. Biol. Lett. **16**, 20200607. (10.1098/rsbl.2020.0607) 33321066 PMC7775973

[7] Zeng Y, Baciadonna L, Davies JR, Pilenga C, Favaro L, Garcia-Pelegrin E. 2024 Bottlenose dolphins (Tursiops truncatus) display gaze alternation and referential communication in an impossible task. Heliyon **10**, e33192. (10.1016/j.heliyon.2024.e33192) 39005890 PMC11239698

[8] Pérez Fraga P, Gerencsér L, Lovas M, Újváry D, Andics A. 2021 Who turns to the human? Companion pigs’ and dogs’ behaviour in the unsolvable task paradigm. Anim. Cogn. **24**, 33–40. (10.1007/s10071-020-01410-2) 32681198 PMC7829225

[9] Pérez Fraga P, Morvai B, Gerencsér L, Lehoczki F, Andics A. 2023 Out-of-reach rewards elicit human-oriented referential communicative behaviours in family dogs but not in family pigs. Sci. Rep. **13**, 811. (10.1038/s41598-022-26503-5) 36690662 PMC9871027

[10] Mendes JW, Resende B, Savalli C. 2021 A review of the unsolvable task in dog communication and cognition: comparing different methodologies. Anim. Cogn. **24**, 907–922. (10.1007/s10071-021-01501-8) 33754284

[11] Heberlein MTE, Turner DC, Range F, Virányi Z. 2016 A comparison between wolves, Canis lupus, and dogs, Canis familiaris, in showing behaviour towards humans. Anim. Behav. **122**, 59–66. (10.1016/j.anbehav.2016.09.023) 27974861 PMC5140004

[12] Gaunet F, Deputte BL. 2011 Functionally referential and intentional communication in the domestic dog: effects of spatial and social contexts. Anim. Cogn. **14**, 849–860. (10.1007/s10071-011-0418-1) 21638003

[13] Gerencsér L, Pérez Fraga P, Lovas M, Újváry D, Andics A. 2019 Comparing interspecific socio-communicative skills of socialized juvenile dogs and miniature pigs. Anim. Cogn. **22**, 917–929. (10.1007/s10071-019-01284-z) 31256339 PMC6834752

[14] Signoret J *et al*. 1975 The behaviour of swine. In Behaviour of domestic animals (ed. ESE Hafez), pp. 295–329. London: Baillière Tindall.

[15] Zonderland JJ, Cornelissen L, Wolthuis-Fillerup M, Spoolder HAM. 2008 Visual acuity of pigs at different light intensities. Appl. Anim. Behav. Sci. **111**, 28–37. (10.1016/j.applanim.2007.05.010)

[16] Groenen MAM *et al*. 2012 Analyses of pig genomes provide insight into porcine demography and evolution. Nature **491**, 393–398. (10.1038/nature11622) 23151582 PMC3566564

[17] Croney CC, Adams KM, Washington CG, Stricklin WR. 2003 A note on visual, olfactory and spatial cue use in foraging behavior of pigs: indirectly assessing cognitive abilities. Appl. Anim. Behav. Sci. **83**, 303–308. (10.1016/s0168-1591(03)00128-x)

[18] Studnitz M, Jensen MB, Pedersen LJ. 2007 Why do pigs root and in what will they root?: a review on the exploratory behaviour of pigs in relation to environmental enrichment. Appl. Anim. Behav. Sci. **107**, 183–197. (10.1016/j.applanim.2006.11.013Get)

[19] Stolba A, Wood-Gush DGM. 1989 The behaviour of pigs in a semi-natural environment. Anim. Sci. **48**, 419–425. (10.1017/s0003356100040411)

[20] Miklósi Á, Kubinyi E, Topál J, Gácsi M, Virányi Z, Csányi V. 2003 A simple reason for a big difference: wolves do not look back at humans, but dogs do. Curr. Biol. **13**, 763–766. (10.1016/s0960-9822(03)00263-x) 12725735

[21] Lehoczki F, Pérez Fraga P, Andics A. 2024 Family pigs’ and dogs’ reactions to human emotional vocalizations: a citizen science study. Anim. Behav. **214**, 207–218. (10.1016/j.anbehav.2024.05.011)

[22] Gisev N, Bell JS, Chen TF. 2013 Interrater agreement and interrater reliability: key concepts, approaches, and applications. Res. Soc. Adm. Pharm. **9**, 330–338. (10.1016/j.sapharm.2012.04.004) 22695215

[23] Pérez Fraga P, Gerencsér L, Andics A. 2020 Human proximity seeking in family pigs and dogs. Sci. Rep. **10**, 20883. (10.1038/s41598-020-77643-5) 33257733 PMC7705753

[24] Savalli C, Ades C, Gaunet F. 2014 Are dogs able to communicate with their owners about a desirable food in a referential and intentional way? PLoS One **9**, e108003. (10.1371/journal.pone.0108003) 25232956 PMC4169500

[25] Zhang L, Needham KB, Juma S, Si X, Martin F. 2021 Feline communication strategies when presented with an unsolvable task: the attentional state of the person matters. Anim. Cogn. **24**, 1109–1119. (10.1007/s10071-021-01503-6) 33797625 PMC8360888

[26] Merola I, Prato-Previde E, Marshall-Pescini S. 2012 Dogs’ social referencing towards owners and strangers. PLoS One **7**, e47653. (10.1371/journal.pone.0047653) 23071828 PMC3469536

[27] Nawroth C, Ebersbach M, von Borell E. 2013 Are juvenile domestic pigs (Sus scrofa domestica) sensitive to the attentive states of humans?--the impact of impulsivity on choice behaviour. Behav. Process. **96**, 53–58. (10.1016/j.beproc.2013.03.002) 23500190

[28] Albiach-Serrano A, Bräuer J, Cacchione T, Zickert N, Amici F. 2012 The effect of domestication and ontogeny in swine cognition (Sus scrofa scrofa and S. s. domestica). Appl. Anim. Behav. Sci. **141**, 25–35. (10.1016/j.applanim.2012.07.005)

[29] Bensoussan S, Cornil M, Meunier-Salaün MC, Tallet C. 2016 Piglets learn to use combined human-given visual and auditory signals to find a hidden reward in an object choice task. PLoS One **11**, e0164988. (10.1371/journal.pone.0164988) 27792731 PMC5085045

[30] Mariti C, Falaschi C, Zilocchi M, Fatjó J, Sighieri C, Ogi A, Gazzano A. 2017 Analysis of the intraspecific visual communication in the domestic dog (Canis familiaris): a pilot study on the case of calming signals. J. Vet. Behav. **18**, 49–55. (10.1016/j.jveb.2016.12.009)

[31] Kittawornrat A, Zimmerman JJ. 2011 Toward a better understanding of pig behavior and pig welfare. Anim. Health Res. Rev. **12**, 25–32. (10.1017/s1466252310000174) 21092389

[32] Passalacqua C, Marshall-Pescini S, Barnard S, Lakatos G, Valsecchi P, Prato Previde E. 2011 Human-directed gazing behaviour in puppies and adult dogs, Canis lupus familiaris. Anim. Behav. **82**, 1043–1050. (10.1016/j.anbehav.2011.07.039)

[33] Topál J, Miklósi Á, Csányi V, Dóka A. 1998 Attachment behavior in dogs (Canis familiaris): a new application of Ainsworth’s (1969) strange situation test. J. Comp. Psychol. **112**, 219–229. (10.1037//0735-7036.112.3.219) 9770312

[34] Gábor A, Pérez Fraga P, Gácsi M, Gerencsér L, Andics A. 2024 Domestication and exposure to human social stimuli are not sufficient to trigger attachment to humans: a companion pig-dog comparative study. Sci. Rep. **14**, 14058. (10.1038/s41598-024-63529-3) 38977716 PMC11231355

[35] Bray EE, Gnanadesikan GE, Horschler DJ, Levy KM, Kennedy BS, Famula TR, MacLean EL. 2021 Early-emerging and highly heritable sensitivity to human communication in dogs. Curr. Biol. **31**, 3132–3136. (10.1016/j.cub.2021.04.055) 34087106

[36] Hare B, Tomasello M. 2005 Human-like social skills in dogs? Trends Cogn. Sci. **9**, 439–444. (10.1016/j.tics.2005.07.003) 16061417

[37] Topál J, Gácsi M. 2012 Lessons we should learn from our unique relationship with dogs: an ethological approach. In Crossing boundaries, pp. 161–186. BRILL. (10.1163/9789004233041_010)

[38] Miklósi Á, Topál J. 2013 What does it take to become ‘best friends’? Evolutionary changes in canine social competence. Trends Cogn. Sci. **17**, 287–294. (10.1016/j.tics.2013.04.005) 23643552

[39] Driscoll CA, Macdonald DW, O’Brien SJ. 2009 From wild animals to domestic pets, an evolutionary view of domestication. Proc. Natl Acad. Sci. USA **106**, 9971–9978. (10.1073/pnas.0901586106) 19528637 PMC2702791

[40] Linhart P, Ratcliffe VF, Reby D, Špinka M. 2015 Expression of emotional arousal in two different piglet call types. PLoS One **10**, e0135414. (10.1371/journal.pone.0135414) 26274816 PMC4537126

[41] Leliveld LMC, Düpjan S, Tuchscherer A, Puppe B. 2016 Behavioural and physiological measures indicate subtle variations in the emotional valence of young pigs. Physiol. Behav. **157**, 116–124. (10.1016/j.physbeh.2016.02.002) 26850291

[42] Briefer EF *et al*. 2022 Classification of pig calls produced from birth to slaughter according to their emotional valence and context of production. Sci. Rep. **12**, 3409. (10.1038/s41598-022-07174-8) 35256620 PMC8901661

[43] Camerlink I, Turner SP. 2013 The pig’s nose and its role in dominance relationships and harmful behaviour. Appl. Anim. Behav. Sci. **145**, 84–91. (10.1016/j.applanim.2013.02.008)

[44] Brown SM, Klaffenböck M, Nevison IM, Lawrence AB. 2015 Evidence for litter differences in play behaviour in pre-weaned pigs. Appl. Anim. Behav. Sci. **172**, 17–25. (10.1016/j.applanim.2015.09.007) 26937060 PMC4768079

[45] Terlouw EMC, Porcher J. 2005 Repeated handling of pigs during rearing. I. Refusal of contact by the handler and reactivity to familiar and unfamiliar humans. J. Anim. Sci. **83**, 1653–1663. (10.2527/2005.8371653x) 15956474

[46] Pérez Fraga P, Andics A, Lehoczki F. 2025 Supplementary material from: Companion pigs alternate orientation between humans and snout-inaccessible targets. Figshare. (10.6084/m9.figshare.c.7987109) PMC12381496 40881983

